# Natural killer cells impede the engraftment of cardiomyocytes derived from induced pluripotent stem cells in syngeneic mouse model

**DOI:** 10.1038/s41598-019-47134-3

**Published:** 2019-07-25

**Authors:** Yuki Nakamura, Shigeru Miyagawa, Shohei Yoshida, Shigemi Sasawatari, Toshihiko Toyofuku, Koichi Toda, Yoshiki Sawa

**Affiliations:** 1 Department of Cardiovascular Surgery, Osaka University Graduate School of Medicine, Suita, Osaka 565-0871 Japan; 2 Department of Immunology and Regenerative Medicine, Osaka University Graduate School of Medicine, Suita, Osaka 565-0871 Japan

**Keywords:** Innate immune cells, Induced pluripotent stem cells

## Abstract

Transplantation of cardiomyocytes derived from induced pluripotent stem cell (iPSC-CMs) is a promising approach for increasing functional CMs during end-stage heart failure. Although major histocompatibility complex (MHC) class I matching between donor cells and recipient could reduce acquired immune rejection, innate immune responses may have negative effects on transplanted iPSC-CMs. Here, we demonstrated that natural killer cells (NKCs) infiltrated in iPSC-CM transplants even in a syngeneic mouse model. The depletion of NKCs using an anti-NKC antibody rescued transplanted iPSC-CMs, suggesting that iPSC-CMs activated NKC-mediated innate immunity. Surprisingly, iPSC-CMs lost inhibitory MHCs but not activating ligands for NKCs. Re-expression of MHC class I induced by IFN-γ as well as suppression of activating ligands by an antibody rescued the transplanted iPSC-CMs. Thus, NKCs impede the engraftment of transplanted iPSC-CMs because of lost MHC class I, and our results provide a basis for an approach to improve iPSC-CM engraftment.

## Introduction

End-stage heart failure is generally characterized by an insufficient number of functional cardiomyocytes (CMs)^1^. Although several clinical efforts involving pharmacological drugs and mechanical devices have been applied for treating end-stage heart failure, the beneficial effects of these maneuver proved to be limited^2^. Thus, transplanting CMs derived from induced pluripotent stem cells (iPSC-CMs) is a promising approach for end-stage heart failure because it can increase the number of functional CMs^3,4^. Indeed, previous studies have demonstrated the beneficial effects of transplanted iPSC-CMs on the cardiac function of ischemic hearts in a mouse model^5,6^ and in a pig model^7^.

For successful cell transplantation, transplanted cells should escape from immune rejection, which mainly occur through major histocompatibility complex (MHC)-mediated acquired immune responses. We have reported that acquired immune responses to transplanted iPSC-CMs in primates decreased dramatically in the MHC class I-matched model compared with the MHC class I-unmatched model^8^. However, previous studies suggested that different immune responses such as with the innate immune system involving natural killer cells (NKCs) may have deleterious effects on transplanted cells even in MHC-matched model^8,9^.

NKCs express inhibitory receptors for MHC class I molecules on target cells. MHC class I molecules, which are expressed on most cells, play a central role in inhibiting NKC-dependent lysis^10,11^. Therefore, MHC class I-negative cells are lysed by NKCs through the “missing-self response^12–14^”. NKCs also express activating receptors such as CD226^15,16^ and natural killer group 2D (NKG2D)^17,18^. The balance of activating and inhibitory signals determines NKC activation against target cells^19^.

In this study, we aimed to investigate the innate immune response against transplanted syngeneic mouse iPSC-CMs (miPSC-CM) after *in vivo* transplantation. Histological analysis in syngeneic model showed large amounts of NKCs infiltrating in the transplants compared with other innate immune cells and T cells, suggesting that NKC-mediated innate immune responses may occur in the transplants. Surprisingly, miPSC-CMs lost inhibitory ligands such as MHC class I molecules, but not activating ligands for CD226 and NKG2D on their surfaces. These results indicated that NKCs play an important role in the survival of transplanted miPSC-CMs through innate immune responses.

## Results

### Cardiomyogenic differentiation of mouse miPSCs and elimination of undifferentiated miPSCs

**Figure 1:**
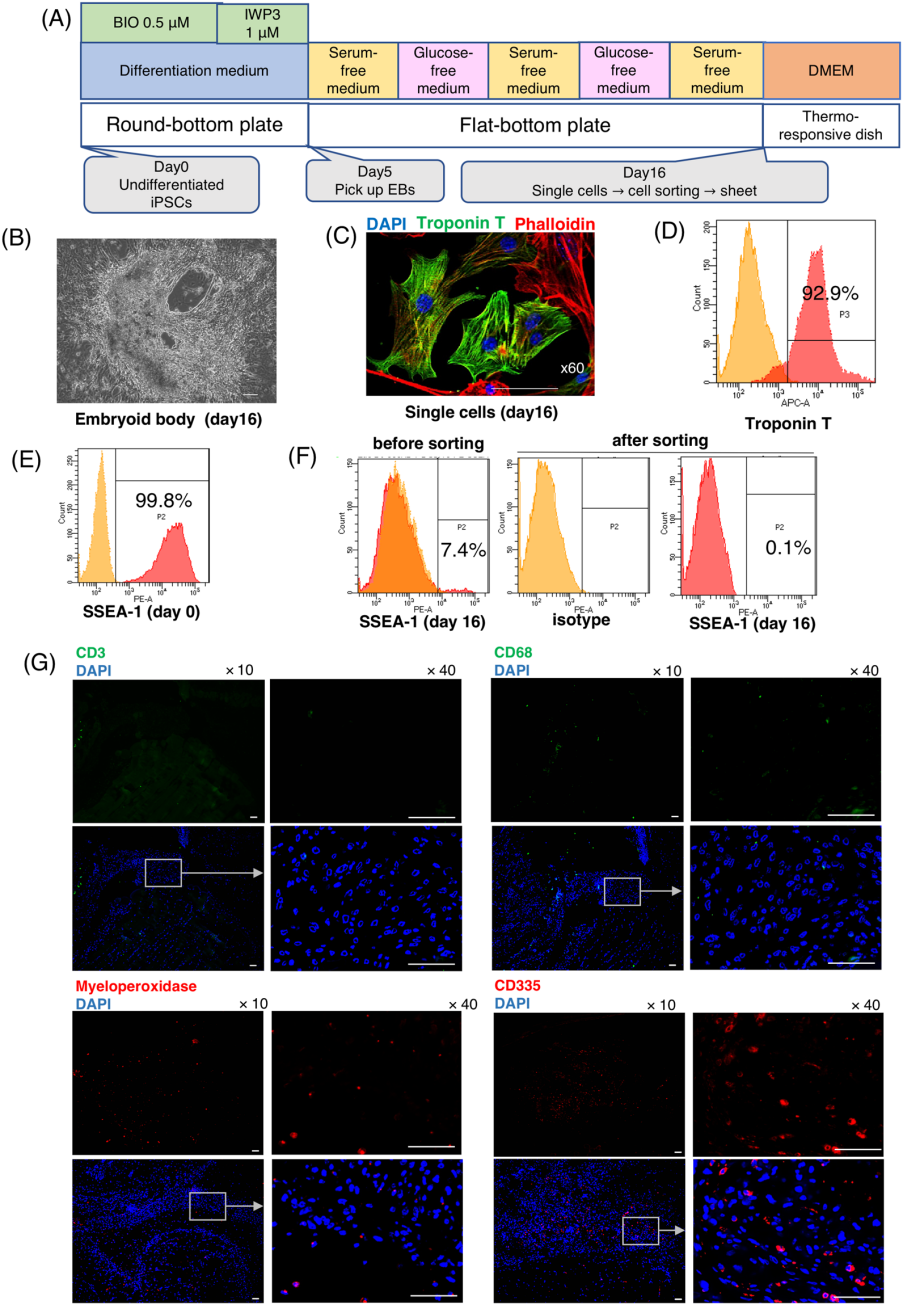
Cardiomyogenic differentiation of murine iPSCs, elimination of undifferentiated iPSCs, and innate immune responses after transplantation of syngeneic iPSC-CMs *in vivo*. (**A**) CM-differentiation protocol. (**B**) Embryoid body of iPSC-CMs at day 16. Scale bar, 100 μm. (**C**) iPSC-CMs at day 16, stained with an anti-troponin T antibody (Alexa Fluor 488), an anti-phalloidin antibody (Alexa Flour 555), and 4′,6-diamidino-2-phenylindole (DAPI). Scale bar, 50 μm. (**D**) Flow cytometry data for iPSC-CMs at day 16, stained with anti-troponin T antibodies or an isotype-matched control. (**E**) Flow cytometry data for undifferentiated iPSCs at day 0 and (**F**) iPSC-CMs before and after cell sorting at day 16, stained with SSEA-1 antibodies or the isotype control. (**G**) Subcutaneous tissue 4 days after iPSC-CM syngeneic transplantation stained with anti-CD3 (Alexa Fluor 488), anti-CD68 (Alexa Fluor 488), anti-myeloperoxidase (Alexa Flour 647) and anti-CD335 (Alexa Flour 555) antibodies, and DAPI. Scale bars: 50 μm.

We used the miPSC line, 959A2-1, generated from C57BL/6 mouse embryonic fibroblasts, which were then treated with neomycin to establish an miPSC line that stably expressed DsRed and luciferase (DsRed-Luciferase-miPSC), as previously described^20^. Cardiomyogenic differentiation of DsRed-Luciferase-miPSCs was induced using a slightly modified culture protocol, according to the schema shown in Fig. 1A. On day 6, cells spontaneously started regular beating and well-beating embryoid bodies of miPSC-CMs were observed on day 16 (Fig. 1B and Movie S1). After 16 days of differentiation, immunohistochemical staining of the beating cell clusters demonstrated that troponin T were labeled in the cytoplasm, indicating the formation of well-aligned sarcomere structures (Fig. 1C). Flow cytometry showed that 66.7 ± 10.6% of the cells were positive for troponin T (Fig. 1D), whereas 3–7% of the cells were positive for stage-specific embryo antigen (SSEA)-1, one of the major cell-surface markers of undifferentiated miPSCs, as assessed by flow cytometry (Fig. 1E). To eliminate undifferentiated miPSCs, SSEA-1-negative miPSC-CMs were isolated with an SH800Z cell sorter (Sony). After sorting, SSEA-1-positive cells were not detected by flow cytometry (Fig. 1F). The percentage of Troponin T-positive cells was maintained, even after sorting (Fig. S1A). Among the miPSC lines, miPSC-CMs representing 60–70% of Toponin T-positive cells were used in the subsequent experiments, similar to a previous study^20^. The SSEA-1-negative miPSC-CMs were seeded in temperature-responsive dishes, and the miPSC-CM sheets (which showed self-beating; Movie S2) were collected at room temperature just before transplantation.

### High-level secretion of chemokines from miPSC-CMs

Immunoassays performed using fluorescently labeled microspheres demonstrated that the miPSC-CM sheet culture supernatant contained high concentrations of several chemokines, such as stromal cell-derived factor 1α (SDF-1α/CXCL12) and monocyte chemotactic protein-1 (MCP-1/CCL2) (Fig. S1B), which are known to affect NKC activity^21^. The iPSC-CM sheet culture supernatant contained low amounts of CCL19, CXCL10, and CXCL11; CXC3L1 and CCL21 were not investigated. A NKC migration assay using the miPSC-CM sheet culture supernatant demonstrated that culture supernatants significantly chemoattracted NKCs, whereas the administration of blocking antibodies against SDF-1α/CXCL12 and MCP-1/CCL2 significantly reduced NKC chemotaxis (Fig. S1C).

### NKCs were recruited into miPSC-CM transplants in a syngeneic model

**Figure 2:**
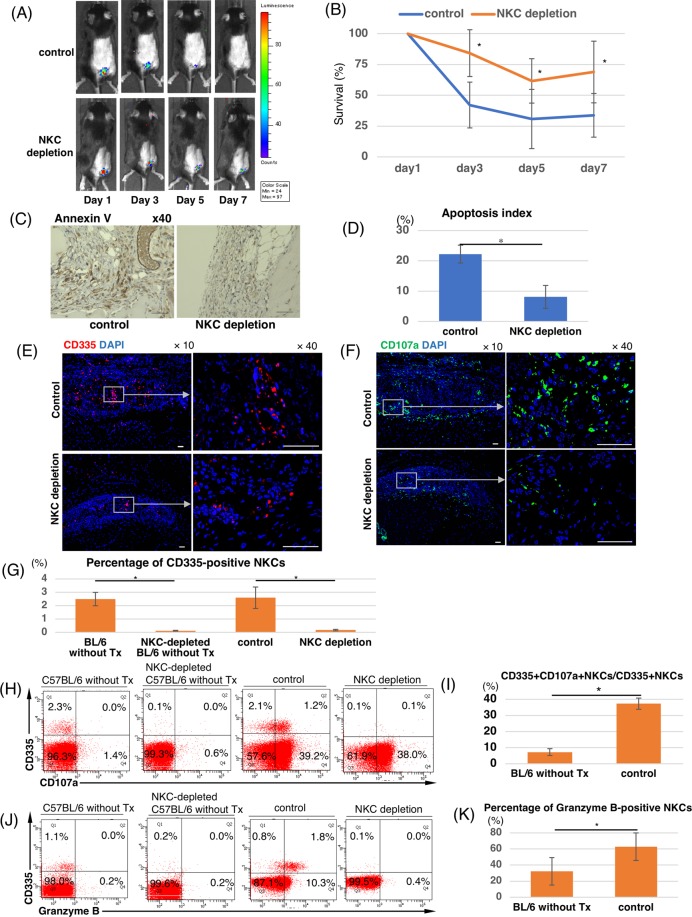
Engraftment of transplanted iPSC-CMs in subcutaneous tissue and activated NKC infiltration into transplants containing iPSC-CMs in an NKC-depleted syngeneic transplantation model. (**A**) Engraftment of transplanted iPSC-CMs was analyzed by BLI in an NKC-depleted model. Representative serial images of the BLI study in one NKC depleted and one control syngeneic mouse. (**B**) The relative quantities of transplanted iPSC-CMs were compared with those at 1 day after transplantation and expressed as the mean ± SD (n = 6, respectively). *p < 0.05. (**C**) Annexin V staining of harvested subcutaneous tissue containing iPSC-CM grafts 7 days after transplantation. Scale bars: 50 μm. (**D**) Semi-quantitative scoring (apoptosis index) calculated by dividing the number of Annexin V-positive cells by the total number of cells in the transplanted sheet 7 days after transplantation. The results are shown as the mean ± SD, *p < 0.05. (**E**) Staining of subcutaneous tissue with DAPI and an anti-CD335 antibody (Alexa Flour 555) at 7 days post-iPSC-CM transplantation in an NKC-depleted model. Scale bars: 50 μm. (**F**) Staining of subcutaneous tissue with DAPI and an anti-CD107a antibody (Alexa Flour 488) at 7 days post-iPSC-CM transplantation in an NKC-depleted model. Scale bars: 50 μm. (**G**) Percentage of CD335-positive NKCs in splenocytes harvested from a C57BL/6 mouse without transplantation and a C57BL/6 mouse at 7 days post-transplantation, with or without NKC depletion and expressed as the mean ± SD (n = 4, respectively). *p < 0.05. (**H**) Flow cytometric detection of CD335 and CD107a expression on splenocytes harvested from C57BL/6 mice without transplantation and C57BL/6 mice at 7 days post-transplantation, with or without NKC depletion. (**I**) Percentage of CD107a-positive NKCs in CD335-positive NKCs present in splenocytes isolated from a C57BL/6 mouse without transplantation and a C57BL/6 mouse at 7 days post-transplantation without NKC depletion; expressed as means ± SD (n = 6, respectively); *p < 0.05. (**J**) Flow cytometric detection of CD335 and Granzyme B on splenocytes harvested from a C57BL/6 mouse without transplantation and from a C57BL/6 mouse at 7 days post-transplantation, with or without NKC depletion. (**K**) Percentage of Granzyme B-positive NKCs in CD335-positive NKCs present in splenocytes isolated from a C57BL/6 mouse without transplantation and a C57BL/6 mouse at 7 days post-transplantation without NKC depletion; expressed as means ± SD (n = 6, respectively); *p < 0.05. BLI; bioluminescence imaging.

To investigate the immunological rejection of transplanted miPSC-CMs in a syngeneic model, we subcutaneously transplanted miPSC-CM sheets into the back of C57BL/6 mice and histologically assessed the infiltration of inflammatory cells into or near the transplants at 4 days post-transplantation. Staining was confirmed using mouse spleens as a positive control (Fig. S2A). Regarding adaptive immune responses, no significant infiltration of CD3-positive lymphocytes was observed in syngeneic model (Fig. 1G), while drastic adaptive immune responses with CD3-positive lymphocytes was observed in allogeneic model using BALB/c mice (Fig. 2B). On the basis of these findings, we decided to use only syngeneic model as control to evaluate the impact of innate immune response on the survival of transplanted iPSC-CMs, because acquired immune response would be affected to their survival in allogeneic model. Regarding innate immune responses, NKCs positive for the cytotoxicity receptor CD335 infiltrated into transplants containing miPSC-CMs, whereas CD68-positive macrophages and myeloperoxidase-positive granulocytes, which were thought to be neutrophils, were only detected at trace levels near the transplants in syngeneic model (Fig. 1G). Therefore, we hypothesized that NKCs may play an important role in immune responses against transplanted miPSC-CMs.

### Recruited NKCs deteriorated miPSC-CMs

To determine whether recruited NKCs deteriorate miPSC-CMs, we transplanted miPSC-CMs into mice lacking NKCs by using anti-NK1.1 antibody. Flow cytometry confirmed that administration of an anti-NK1.1 antibody successfully depleted NKCs in the spleens of treated mice (Fig. S3A). The miPSC-derived cardiac tissue sheets were transplanted subcutaneously into control (syngeneic model; n = 6) and anti-NK1.1 antibody-treated C57BL/6 mice (NK-depleted syngeneic model; n = 6). Quantitative bioluminescence imaging (BLI) demonstrated that the luminescence intensity of miPSC-CMs in the NKC-depleted syngeneic mice at post-transplantation day 7 was significantly higher than those in control mice (68.9 ± 25.0% vs. 33.7 ± 17.7%, p < 0.05) (Fig. 2A,B). We also observed more surviving DsRed-positive and Troponin T-positive iPSC-CMs at post-transplantation day 7 in NKC-depleted mice than in control mice (Fig. S3B). Moreover, the apoptosis index, which was calculated by dividing the number of Annexin V-positive cells by the total number of cells in each transplanted sheet, was significantly higher in control mice vs. NKC-depleted mice (22.2 ± 2.9% vs. 8.1 ± 3.8%, p < 0.05) (Fig. 2C,D). These results indicated that recruited NKCs impeded the engraftment of miPSC-CM transplants in the syngeneic transplantation model.

In the transplants, multifocal infiltration of CD335-positive NKCs in the grafts was histologically observed with the control model. Only slight NKC infiltration was observed around the grafts with the NKC-depleted model (Fig. 2E). Additionally, a functional marker for NKC activity, CD107a-positive cells in and around the grafts were observed more with the control model than in the NKC-depleted model (Fig. 2F). Although the percentage of CD335-positive NKCs in the spleen was not significantly higher in control mice compared with mice without miPSC-CM transplantation (Fig. 2G), the percentage of CD107a-positive NKCs in control mice was significantly higher than that in mice without miPSC-CM transplantation (37.4 ± 3.5% vs. 7.2 ± 2.1%, p < 0.05) (Fig. 2H,I). Additionally, the percentage of NKCs positive for Granzyme B was significantly higher in control mice than in mice without miPSC-CM transplantation (62.9 ± 3.1 vs. 31.6 ± 17.1%, p < 0.05) (Fig. 2J,K). These results indicated that recruited NKCs were activated after the transplants of miPSC-CMs.

### Reduced expression of MHC class I molecules and the existence of NKC-activating ligands on miPSC-CMs

**Figure 3:**
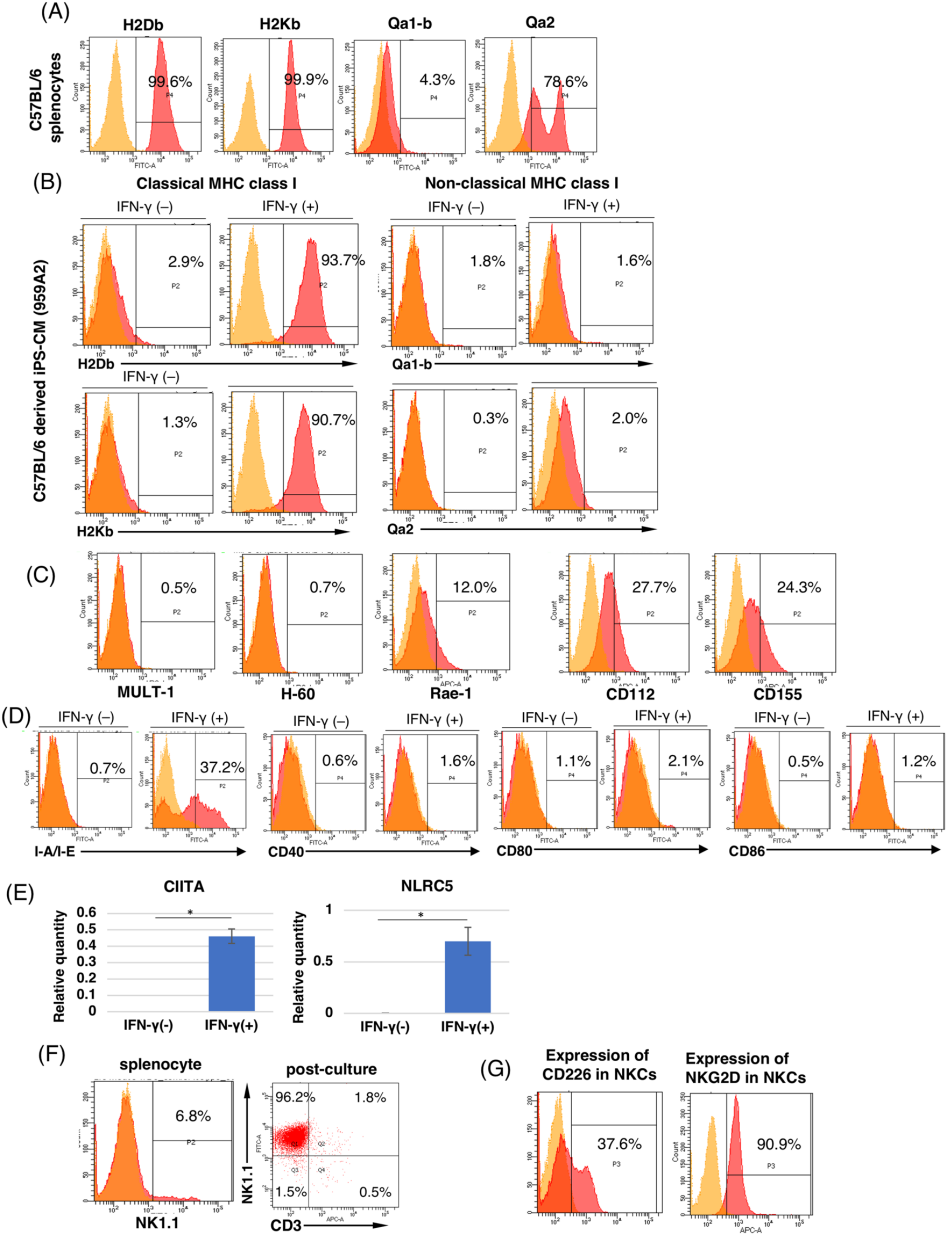
Expression analysis of NKC ligands on iPSC-CMs with or without IFN-γ treatment and expression of NKC-activating receptors on purified NKCs before coculture. (**A**) Flow cytometric detection of MHC class I molecule expression on C57BL/6 splenocytes using antibodies against H2Db, H2Kb, Qa1-b, and Qa2, or isotype-matched control antibodies. (**B**) Flow cytometric detection of MHC class I molecule expression on iPSC-CMs with or without IFN-γ treatment. The iPSC-CMs were stained with antibodies against H2Db, H2Kb, Qa1-b, and Qa2, or isotype-matched control antibodies. (**C**) Flow cytometric detection of NKC-activating ligands on iPSC-CMs at day 16. The iPSC-CMs were stained with antibodies against MULT1, H-60, RAE-1, CD112, and CD155, or isotype-matched control antibodies. (**D**) Flow cytometric detection of MHC class II (I-A/I-E) molecules on iPSC-CMs with or without IFN-γ treatment, stained with anti-I-A/I-E, CD40, CD80, and CD86 antibodies or isotype-matched control antibodies. (**E**) mRNA expression of CIITA and NLRC5 in iPSC-CMs with or without IFN-γ treatment, as measured by real-time PCR. The results are expressed relative to those of C57BL/6 splenocytes as means ± SD (n = 3, respectively); *p < 0.05. (**F**) Flow cytometric detection of NK1.1 and CD3 expression on cultured NKCs purified from C57BL/6 splenocytes, stained with antibodies against NK1.1 and CD3, or isotype-matched control antibodies. (**G**) Flow cytometric detection of NKC-activating receptors on cultured NKCs purified from C57BL/6 splenocytes, stained with antibodies against CD226 and NKG2D, or isotype-matched control antibodies.

To determine how NKCs were recruited into miPSC-CM transplants, we investigated the expression of NKC ligands on miPSC-CMs using flow cytometry. Regarding inhibitory ligands of NKCs, such as MHC class I, flow cytometry demonstrated that classical MHC class I expression on miPSC-CMs was significantly lower than that on native splenocytes derived from C57BL/6 mice (splenocytes vs. miPSC-CMs; H2Db, 98.9 ± 0.6% vs. 2.1 ± 0.6%; H2Kb, 96.4 ± 3.3% vs. 0.7 ± 0.4%; P < 0.05) (Fig. 3A,B). Lower expression of Q2 was also detected on miPSC-CMs, compared with native splenocytes (splenocytes vs. miPSC-CMs; Q2, 38.6 ± 22.0% vs. 0.3 ± 0.2%; P < 0.05) (Fig. 3A,B). Regarding activating ligands of NKCs, we found that CD112 and CD155 (which are ligands of the activating receptor CD226) were expressed on miPSC-CMs (CD112, 45.4 ± 18.2%; CD155, 29.1 ± 5.0%). RAE-1, which is an NKG2D ligand, was expressed at low levels on miPSC-CMs (Figs 3C and S4). These results indicated that preferential expression of activating ligands for NKCs were expressed on miPSC-CMs.

### NKC-mediated immune responses were determined by the expression pattern of surface ligands on miPSC-CMs

**Figure 4:**
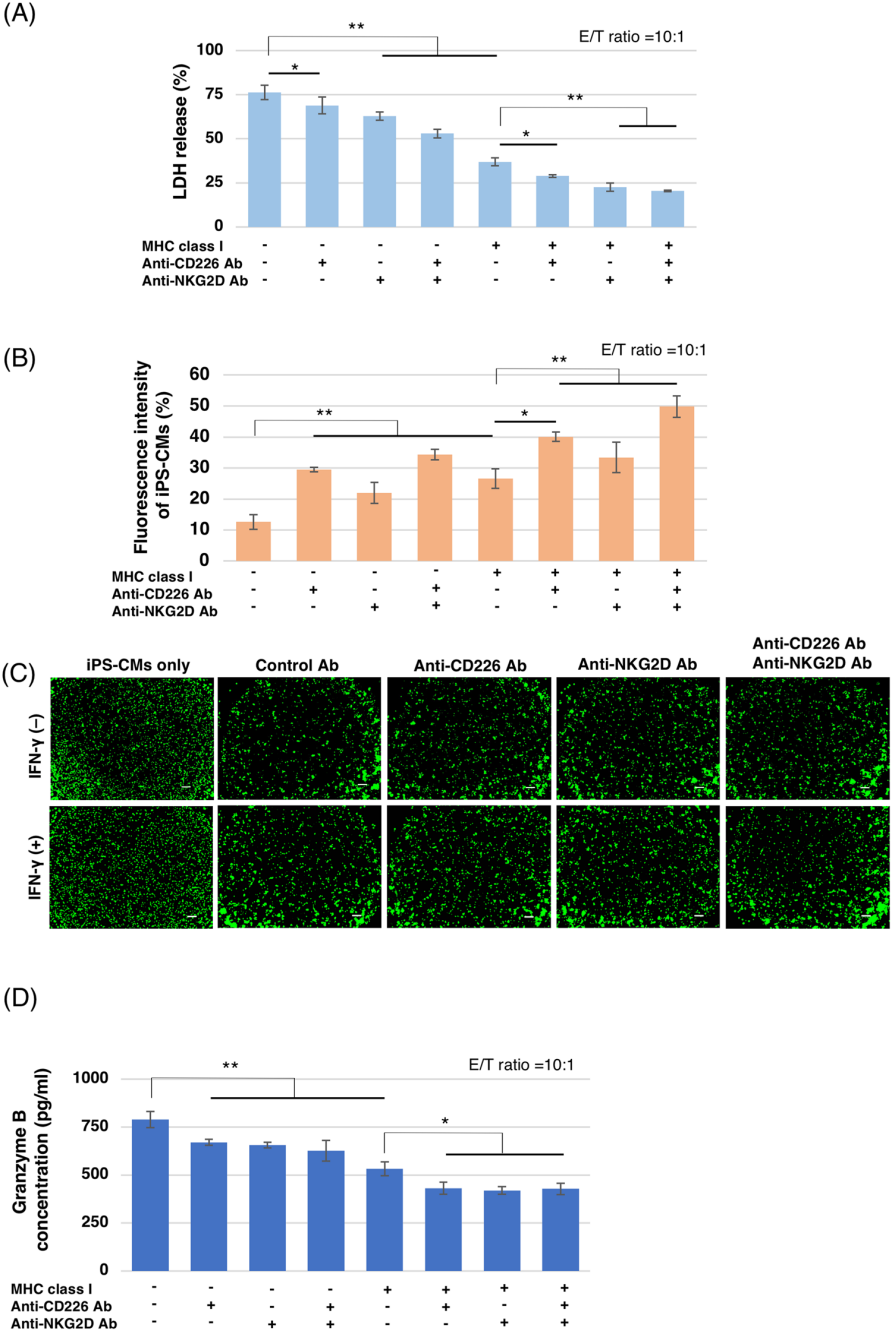
The cytotoxic effect of NKCs on iPSC-CMs *in vitro*. (**A**) The percentage of LDH release in supernatants of co-cultured iPSC-CMs and NKCs compared with that of 0.5% Triton X-treated controls; expressed as means ± SD (n = 3, respectively); **p < 0.001, *p < 0.05. (**B**) The percentage of fluorescence intensities of iPSC-CMs labeled with CAM compared with that of 0.5% Triton X-treated control; expressed as means ± SD (n = 3, respectively); **p < 0.0001, *p < 0.05. (**C**) Images of viable iPSC-CMs labeled with CAM after co-culture. Scale bar: 200 μm (**D**) The concentration of Granzyme B in the supernatants of co-cultures iPSC-CMs and NKCs; expressed as means ± SD (n = 3, respectively); **p < 0.0001, *p < 0.01.

To determine whether NKC-mediated immune responses were induced by NKC-ligand expression on miPSC-CMs, the cytotoxicity of miPSC-CMs incubated with purified NKCs from C57BL/6 mice was examined by performing lactate dehydrogenase (LDH)-release assays and calcein AM (CAM) assays. When miPSC-CMs were incubated with IFN-γ, the expression of classical MHC class I on miPSC-CMs was upregulated significantly, compared with untreated miPSC-CMs. In contrast, the expression of non-classical MHC class I on miPSC-CMs was not upregulated by IFN-γ treatment (Fig. 3B). MHC class II expression on miPSC-CMs was also upregulated by IFN-γ treatment, compared with untreated miPSC-CMs (Fig. 3D). Additionally, the expression of CIITA and NLRC5, which are key transcriptional activators of MHC class I and class II in miPSC-CMs, was significantly upregulated by IFN-γ treatment (Fig. 3E). Flow cytometry showed that 96.1 ± 2.6% of the cultured NKCs extracted from C57BL/6 mouse spleens were positive for NK1.1 and negative for CD3 (Fig. 3F). Among these purified NKCs, 37.6% were positive for CD226 and 82.4% were positive for NKG2D (Fig. 3G). After co-culturing NKCs and miPSC-CMs with or without IFN-γ treatment, the expression of classical MHC class I significantly decreased the release of LDH in miPSC-CMs (Fig. 4A, and S5A). This treatment also significantly increased the cell viability in miPSC-CMs (Figs 4B,C and S5B) and decreased Granzyme B levels in the co-culture supernatant (Fig. 4D). These results indicated that the loss of classical MHC class I in miPSC-CMs augmented NKC-mediated toxicity to miPSC-CMs.

The administration of blocking antibodies against CD226 or NKG2D significantly decreased LDH release (Figs 4A and S5C,D). These antibodies also increased the viability of miPSC-CMs, as determined in CAM assays (Figs 4B,C and S5E,F) and decreased levels of Granzyme B (Fig. 4D). These results indicated that activating NKC ligands also augmented NKC-mediated immune responses. Together, these data suggest that NKC-mediated immune responses were induced by both NKC ligand expression and the loss of classical MHC class I on miPSC-CMs.

### Upregulation of MHC-class I and blocking CD226 and NKG2D reduced NKC infiltration into transplants and miPSC-CM necrosis

**Figure 5:**
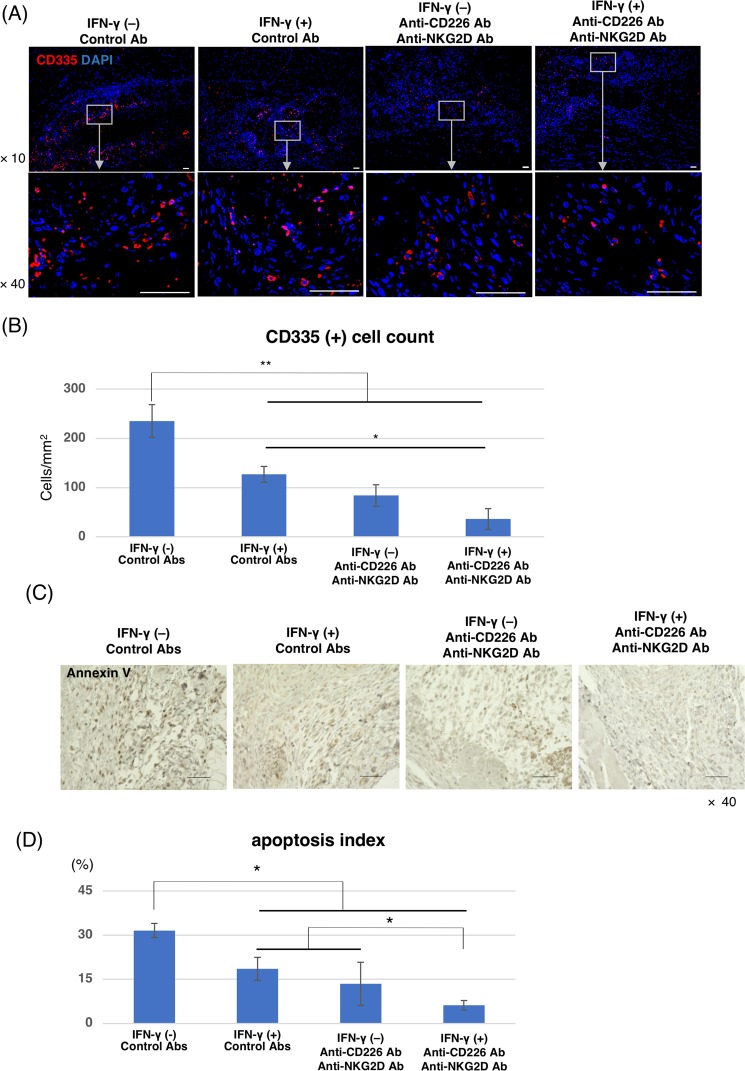
NKC infiltration and apoptotic cells in transplants containing iPSC-CMs. (**A**) Subcutaneous tissue 7 days after iPSC-CM transplantation stained with an anti-CD335 antibody (Alexa Flour 555) and DAPI. Scale bars: 50 μm. (**B**) Semi-quantitative scoring for the number of the CD335-positive cells in the grafts 7 days after transplantation. The results are shown as means ± SD (n = 6, respectively); **p < 0.001, *p < 0.01. (**C**) Annexin V staining of harvested subcutaneous tissue containing iPSC-CM grafts 7 days after transplantation. Scale bars: 50 μm. (**D**) Semi-quantitative scoring calculated by dividing the number of Annexin V-positive cells by the total number of cells in the transplanted sheet 7 days after transplantation. The results are shown as means ± SD (n = 6, respectively); *p < 0.01.

Finally, we investigated whether NKC-related responses after miPSC-CM transplantation could be suppressed by inducing MHC class I on miPSC-CMs and blocking pathway activation via CD226 and NKG2D. The miPSC-CM sheets were generated in the presence or absence of IFN-γ treatment. Both types of miPSC-CM sheets were transplanted subcutaneously into C57BL/6 mice, which were treated with or without a cocktail of blocking antibodies against CD226 and NKG2D (MHC– control group, MHC+ control group, MHC– Ab group, and MHC+ Ab group; n = 6 in each group). Immunohistochemistry demonstrated that the number of NKCs infiltrating the transplants was significantly suppressed by MHC class I upregulation by IFN-γ or by blocking activation pathway via CD226 and NKG2D (MHC– control group, MHC + control group, MHC– Ab group, and MHC+ Ab group values: 235 ± 33 cells/mm^2^, 127 ± 15 cells/mm^2^, 84 ± 22 cells/mm^2^, and 36 ± 21 cells/mm^2^, respectively) (Fig. 5A,B). In addition, the apoptosis index in the transplants was significantly reduced by these two treatments (MHC– control group vs. MHC + control group, 31.6 ± 2.4% and 18.6 ± 3.9%; and MHC– Ab group vs. MHC + Ab group, 13.5 ± 7.3% and 6.2 ± 1.6%, respectively) (Fig. 5C,D). Therefore, MHC class I expression on miPSC-CMs and blocking activating pathways may improve the engraftment of miPSC-CMs after transplantation.

## Discussion

In this study, remarkable NKC infiltration into miPSC-CM transplants and systemic NKC activation were observed after transplantation of miPSC-CMs in MHC-matched mice. Administering an anti-NK1.1 antibody to eliminate endogenous NKCs significantly increased the numbers of engrafted miPSC-CMs in MHC-matched mice. Thus, NKC-related innate immune responses impeded the engraftment of miPSC-CMs. In agreement with this finding, miPSC-CMs lost cell-surface expression of MHC class I, which is required for MHC-dependent NKC inhibition.

Previous findings demonstrated that differentiated iPSCs, such as retinal pigment epithelial cells and vascular endothelial cells, showed similar levels of MHC class I expression compared with their native cells^22,23^; consequently, *in vitro*-differentiated cells acquired resistance against autologous NKCs^24^. It is thus quite important to determine how miPSC-CMs lose MHC class I expression. Constitutive and induced transcription of the MHC-I and MHC-II genes is mediated by a set of conserved regulatory elements in their promoters and interacting transcription factors of which the SXY-module is shared by both sets of genes^25,26^. The SXY module is cooperatively bound by a multi-protein complex. Among them, the class II transactivator (CIITA) and NOD-, LRR- and CARD-containing 5 (NLRC5) play a major role in IFN-γ-induced MHC expression. In miPSC-CMs, IFN-γ exposure induced both CIITA and NLRC5, resulting in MHC class I and II expression^26^. This result suggests that the IFN-γ–CIITA and IFN-γ–NLRC5 axes promote MHC expression in miPSC-CMs. Together with the finding that CIITA and NLRC5 expression was not induced in miPSC-CMs during differentiation from immature miPSCs to miPSC-CMs, it is likely that the inhibitory pathway could constrain CIITA- and NLRC5-dependent transcriptional regulation of the MHC gene. Transcriptional activation of MHC genes also involves modulation of covalent histone modification and chromatin remodeling^27–29^. Thus, cell type-specific expression of MHC in differentiated miPSCs suggests that chromosomal re-modeling may take place during differentiation.

NKC activation against miPSC-CMs is mutually regulated by the characteristic expression of NKC ligands on miPSC-CMs including inhibitory ligands (such as MHC class I) and activating ligands (such as NKG2D and CD226). Lower expression of MHC class I molecules was also found in differentiated CMs derived from macaque iPSCs and embryonic stem (ES) cells^8,24^. *In vitro* syngeneic co-culture experiments demonstrated that miPSC-CM lysis by NKCs was suppressed by IFN-γ-induced MHC class I expression. In contrast, through the activating ligands, mouse and human iPSC and ES cells were killed by NKCs^30–33^. The lysis of mouse iPSC-CMs by syngeneic NKCs decreased in blocking experiments performed with anti-CD226 and anti-NKG2D antibodies. These effects of antibodies on miPSC-CM survival were independent of the expression of classical MHC class I, suggesting that inhibition of activating signals via CD226 and NKG2D were essential for miPSC-CMs to protect against NKC attack. In addition to the direct interaction between miPSC-CMs and NKCs, miPSC-CM sheet culture supernatants contained high concentrations of SDF-1α/CXCL12 and MCP-1/CCL2, which stimulate the migration and cytotoxicity of NKCs, as determined by performing *in vitro* chemotaxis assays^34^. Activated human NKCs have been found to migrate in response to known ligands for SDF-1α/CXCL12^21,35–37^. In contrast, several studies reported the effects of MCP-1/CCL2 on resting NKC cytotoxicity^38–40^. Thus, SDF-1α/CXCL12 may recruit NKCs to transplants and MCP-1/CCL2 may contribute to the activation of NKCs after transplantation.

The heterogeneity of mature mouse NKC can be represented by four subsets on the basis of CD27 and CD11b expression^41^. NKC differentiation has been shown to proceed from CD27^hi^ CD11b^lo^ through CD27^hi^ CD11b^hi^ and ultimately to CD27^lo^ CD11b^hi41^. The cytotoxic NKC subset is mainly CD27^lo^ CD11b^hi^ NKC, and this subset accounts for more than 90% of NK cells from peripheral blood^42^. In this study, we focused on NKC with a natural cytotoxicity receptor (NCR), such as CD335. CD335 is a natural cytotoxicity receptor (NCR) and represents a novel NK cell-specific molecule involved in NK cell activation that has been implicated in NK cell-mediated lysis of several autologous target cell types^43,44^. We did not analyze NKC differentiation in transplanted mice by measuring CD27 and CD11b; however, we suspected that CD27^lo^ CD11b^hi^ NKCs may be major mediators of the NKC-related immune response after miPSC-CM transplantation. Further investigations would be required to determine the subset of NKCs that is responsible for this activity.

For clinical applications, CMs derived from MHC-homozygous iPSCs are supposed to be mostly transplanted to MHC-heterozygous recipients using banked iPSC lines from healthy donors^45^. Although the induced expression of classical MHC class I on miPSC-CM may be an effective treatment for inhibiting cytolysis by NKCs after transplantation in syngeneic models, it remains unclear whether IFN-γ treatment of iPSC-CMs would suppress the cytotoxicity of NKCs in a homo–hetero model. Following transplantation of iPSC-derived dopamine neurons, which weakly expressed MHC class I, NKC infiltration into transplants did not significantly differ between homo-to-hetero MHC-matched and MHC-mismatched settings^46^. However, the heterozygous MHC NKCs scarcely showed specific lysis to homozygous MHC iPSC-derived T cells and endovascular cells with significant MHC class I expression^23^. Therefore, we suspect that IFN-γ treatment of iPSC-CMs may contribute to the suppression of NKCs even in a homo-to-hetero setting, using human iPSCs.

The evaluation of engraftment by subcutaneous graft transplantation is a limitation of our study; however, previous studies reported that immunological responses to iPSC-CM after subcutaneous transplantation and transplantation into cardiac tissues or surfaces were similar. This similarity was confirmed in both mouse^20^ and macaque^8^ models of iPSC-CM transplantation. Moreover, in our previous study, transplanted iPSC-CMs were similarly engrafted or rejected in these transplantation models in subcutaneous tissue as compared with cardiac tissue^8^. Together with these findings, we believe that similar results in our study could be obtained if iPSC-CM were transplanted into cardiac tissue.

In conclusion, the engraftment of miPSC-CMs after transplantation was impeded by innate immune responses, which were induced by NKC activation due to lower expression of MHC class I and the existence of activating ligands for CD226 and NKG2D on miPSC-CMs in the mouse syngeneic transplantation model. Further studies are warranted to elucidate the optimal immunosuppressive treatment including NKC-related innate immune rejection for the clinical application of cell transplantation therapy using human iPSC-CMs.

## Materials and Methods

All animal experiments were performed according to the Guide for the Care and Use of Laboratory Animals published by the National Institutes of Health. The institutional Ethics Review Committee for Animal Experimentation of the Osaka University Graduate School of Medicine approved all experimental protocols.

### Cell culture, cardiomyogenic differentiation, and sheet generation

We treated the 959A2-1 miPSC line (generated from C57BL/6 mouse embryonic fibroblasts) with neomycin to establish an miPSC line that stably expressed DsRed and luciferase (DsRed-Luciferase-miPSC), as previously described^20^. DsRed-Luciferase-miPSCs were cultured in ESGRO complete PLUS Clonal Grade Medium (Merck Millipore). Cardiomyogenic differentiation was induced as previously reported^5,20,47,48^, with slight modifications. To generate embryoid bodies (EBs), 3000 cells were resuspended in 100 μL aliquots of differentiation medium [DM; Dulbecco’s Modified Eagle’s Medium (DMEM; Nacalai Tesque) containing 15% fetal bovine serum (FBS; Biofill), 100 mmol/L non-essential amino acids (NEAA; Invitrogen), 2 mmol/L L-glutamine (Invitrogen), and 0.1 mmol/L 2-mercaptoethanol (Invitrogen)] containing 0.2 mmol/L 6-bromoindirubin- 39-oxime (BIO; a glycogen synthase kinase-3b inhibitor, to activate the Wnt-signaling pathway) (Calbiochem), and cultured in 96-well Corning Costar Ultra-Low attachment multiwell plates (Sigma–Aldrich) to activate the Wnt-signaling pathway. The effect of BIO was neutralized by adding an inhibitor of Wnt production 3 (IWP-3) at 1.0 μmol/L on day 3, and each EB was transferred to a flat-bottom plate for adhesion culture on day 5. On days 6, 10, and 14, culture medium was exchanged for serum-free Modified Eagle’s Medium (MEM; Invitrogen) with insulin transferrin-selenium-X (Invitrogen). On days 8 and 12, culture medium was exchanged for Glucose-free DMEM (no glucose, no pyruvate, Invitrogen) supplemented with 4 mmol/L lactic acid (Wako Pure Chemical) for the purification of cardiomyocytes. On day 16, the cell clusters were dissociated. To eliminate undifferentiated iPSCs, SSEA-1-negative iPSC-CMs were isolated with an SH800Z cell sorter (Sony) and seeded on thermoresponsive dishes (2–2.5 × 10^6^ CMs/well; Upcell; CellSeed), and incubated at 37 °C for 2 to 3 days. They were then transferred to 20 °C until the cells detached spontaneously to form scaffold-free cell sheets before transplantation was performed.

### Transplantation of iPSC-CM sheets

Adult male C57BL/6 (6–9-weeks old, 22–27 g) were generally anesthetized via isoflurane inhalation, and the DsRed-Luciferase-miPSC-CM sheets were subcutaneously transplanted into the backs of mice.

### *In vivo* depletion of NKCs

An anti-NK1.1 (PK136) Ab and a control mouse IgG Ab were purchased from Bio X Cell and Sigma–Aldrich, respectively. mAbs were resuspended in phosphate-buffered saline (PBS). Recipient mice received 400 μg anti-NK1.1 mAb intraperitoneally (i.p.) on days −1, 1, 3, and 5 post-transplantation to deplete NKCs in the mice.

### Immunohistochemical staining and histological analysis

All primary antibodies used for immunohistochemical staining and histological analysis are listed in Table S1A. miPSC-CMs were fixed with 4% paraformaldehyde and labeled with primary antibodies such as anti-cardiac troponin T (Abcam) or anti-phalloidin (Life technologies) (Table S1A), followed by secondary antibodies such as Alexa Fluor 488- or Alexa Fluor 555-conjugated goat or donkey anti-mouse or anti-rabbit antibodies (ThermoFisher Scientific). Nuclei were counterstained with Hoechst33342 (Dojindo). The cells were assessed using a confocal microscope (FLUOVIEW FV10i; Olympus).

Paraffin sections fixed with 4% paraformaldehyde phosphate buffer were used for hematoxylin and eosin staining, and immunohistochemical staining. Samples were labeled with primary antibodies against CD68 (Abcam), CD3 (Abcam), Myeloperoxidase (Abcam), CD335 (BioLegend) and CD107a (Abcam), followed by incubation with secondary antibodies such as Alexa Fluor 488- or Alexa Fluor 555-conjugated goat or donkey anti-mouse or anti-rabbit antibodies (ThermoFisher Scientific). Nuclei were counterstained with Hoechst 33342 (Dojindo). The samples were observed under a BZ 9000 fluorescence microscope (Keyence).

Moreover, samples were microwaved in Target Retrieval Solution (pH 9.0; Dako), treated sequentially with a rabbit anti-mouse Annexin V antibody (Abcam) and a horseradish peroxidase-labeled goat anti-rabbit immunoglobulin, and finally treated with 3,3′-diaminobenzidine. The apoptosis index was calculated by dividing the number of Annexin V-positive cells by the total number of cells in the transplanted sheet, which were observed under a BZ 9000 fluorescence microscope (Keyence) and analyzed using the Dynamic Cell Count function of the BZ-II Analyzer Software (Keyence) in at least 5 hot spots.

### Flow cytometry

All primary antibodies used for flow cytometry are listed in Table S1A. Cultured iPSC-CMs were dissociated with 0.25% trypsin-EDTA for 6 min at 37 °C and labeled with fluorescently conjugated antibodies against H2Db (Abcam), H2Kb (BioLegend), Qa1-b (Novus), Qa2 (Abcam), and SSEA-1 (BioLegend) for 30 min at 4 °C. Dissociated iPSC-CMs were labeled with antibodies against MULT-1 (R&D Systems), Rae-1 (R&D Systems), H60 (R&D Systems), CD112 (R&D Systems), or CD155 (R&D Systems), followed by fluorophore-conjugated secondary antibodies. iPSC-CMs were also labeled with antibodies against cardiac troponin T (BD Pharmingen) after fixation using BD Cytofix Fixation Buffer (Becton Dickinson), followed by incubation with fluorescently conjugated secondary antibodies. Spleens were harvested from C57BL/6 mouse and homogenized, followed by suspension in PBS. The suspended homogenate was then filtered with a 40-*𝜇*m nylon filter (Corning), centrifuged, and incubated with red blood cell lysing buffer (Santa Cruz) for 3 min at room temperature to lyse the red blood cells. After centrifugation, the splenocytes were washed and labeled with fluorescently conjugated antibodies against CD335 (BioLegend), NK1.1 (BioLegend), and CD107a (BioLegend) for 30 min at 4 °C and analyzed using a FACSCanto II flow cytometer (Becton Dickinson). Splenocytes were labeled with antibodies against Granzyme B (BioLegend) after fixation and permeabilization using Foxp3/Transcription Factor Staining Buffer Set (Thermo Fisher Scientific). Samples were assessed using the FACSCanto II system (Becton Dickinson), and data were analyzed using Diva software (Becton Dickinson).

### Luciferase assay and BLI

To evaluate the quantity of transplanted iPSC-CMs, BLI was performed using the IVIS Lumina II instrument (PerkinElmer), as previously described^20^, and the luminescence intensities of the iPSC-CMs were measured. Five minutes before measuring luminescence, RediJect D-Luciferin Ultra Bioluminescence Substrate (PerkinElmer) was injected intraperitoneally (150 mg/kg body weight). The integration time was fixed at 8 min for each image. All images were analyzed with Living Image Software (PerkinElmer).

### LDH-release assay

After splenocytes derived from C57BL/6 were obtained as described above, NKCs were isolated from splenocytes by magnetic-activated cell sorting using negative-selection kits (NK Cell Isolation Kit II, Miltenyi Biotec) and cultured using ALyS505NK-EX medium (Cell, Science & Technology Institute Inc.) with 1000 U/ml mouse IL-2 (BioLegend) for two weeks. iPSC-CMs were treated with 100 ng/ml IFN-γ (R&D Systems) for 48 h. LDH-release assays were performed using an LDH Cytotoxicity Detection Kit (Takara Bio). Cultured NKCs were washed and incubated with blocking antibodies against DNAM-1 (CD226) (Thermo Fisher Scientific) and NKG2D (BioLegend) for 90 min at room temperature. After washing, NKCs were cocultured with iPSC-CMs in a 96-well plate for 6 h. After incubation in the dark for 30 min at room temperature, the absorbance at 490 nm was recorded with a microplate reader (DS Pharma Biomedical), with a reference wavelength of 600 nm. The experiments were performed in triplicate. LDH release was determined as the percentage of LDH released compared with that of the 0.5% Triton X-treated control.

### Cell viability assay

Cell viability of iPSC-CMs after co-culture with NKCs was analyzed using the Terascan VPC system (Minerva Tech), as described previously^49,50^. iPSC-CMs were labeled with CAM (Dojindo) diluted from a 1 mM stock solution in dimethyl sulfoxide for 30 min at 37 °C. The labeled cells were then incubated with NKCs for 4 h. Cells were then washed twice in Dulbecco’s modified Eagle’s medium (DMEM) and adjusted to 2 × 10^5^ cells/ml in DMEM supplemented with 10% FBS. CAM-labeled iPS-CMs and NKCs incubated with neutralizing antibodies (prepared as described above) were cocultured in a 96-well half-area plate (Costar) and incubated in the dark for 30 min at 37 °C. The fluorescence intensity was measured before and after the 4-h culture, and the specific cell viability was calculated as the percentage of fluorescence intensity compared with that of the 0.5% Triton X-treated control.

### Chemokine and Granzyme B measurements

The iPS-CM sheet culture supernatants were collected and analyzed by performing immunoassays based on fluorescently labeled microspheres to measure chemokine production with the Bio-Plex suspension array system (Bio-Rad). Granzyme B levels were measured in the co-culture supernatants of iPS-CMs and NKCs using a Mouse Granzyme B Coated ELISA Kit (Thermo Fisher Scientific), according to the manufacturer’ protocol.

### Real-time polymerase chain reaction (PCR) experiments

Total RNA was extracted using an RNeasy Kit (Qiagen), and cDNA was synthesized using a SuperScript VILO cDNA Synthesis Kit (Thermo Fisher Scientific). Real-time PCR was performed on a Viia7 real-time PCR system (Thermo Fisher Scientific), using TaqMan PCR master mix. The following genes were analyzed using TaqMan gene expression assays (Thermo Fisher Scientific): Ciita (Mm00482914_m1) and Nlrc5 (Mm01243039_m1). Relative gene-expression levels were calculated using the ΔΔCt method, with normalization to glyceraldehyde-3-phosphate dehydrogenase (Mm00482914_m1) expression (Table S1B).

### NKC migration assay

NKCs were labeled with CAM (Dojindo, Kumamoto) as described above. After incubation with blocking antibodies against SDF-1α/CXCL12 (Abcam) and MCP-1/CCL2 (Abcam) for 90 min at room temperature, the iPSC-CM sheet culture supernatants were placed in the bottom wells of a Transwell migration plate (Costar Corning). CAM-labeled NKCs were added to the upper wells, and plates were incubated for 90 min 37 °C to allow the cells to migrate. The fluorescence intensity was recorded with a microplate reader (DS Pharma Biomedical) with an excitation wavelength of 490 nm and an emission wavelength 520 nm. Experiments were performed in triplicate. The migrated cells were quantified by measuring the fluorescence intensity of the bottom wells, and the chemotaxis of NKCs was calculated as the percentage of the fluorescence intensity compared with that of the control well, in which NKCs were added to the bottom wells.

### Transplantation of IFN-γ-treated iPSC-CMs and administration of an antibody cocktail including CD226- and NKG2D-blocking antibodies

iPSC-CMs were treated with 100 ng/ml IFN-γ while generating iPSC-CM sheets on thermoresponsive dishes (2–2.5 × 10^6^ CMs/well; Upcell; CellSeed). Mice were administered blocking antibodies against CD226 (Thermo Fisher Scientific) and NKG2D (BioLegend), or control mouse and hamster IgG Abs (Sigma–Aldrich and BioLegend). mAbs were resuspended in PBS. Recipient mice received 100 μg anti-CD226 mAb and 100 *μ*g anti-NKG2D mAb i.p. on days −1, 1, 3, and 5 days post-transplantation to suppress NKCs in mice.

### Semi-quantitative scoring of the number of CD335-positive cells in the grafts

CD335-positive cells in the grafts were counted in the fluorescently stained samples. The numbers of CD335-positive cells were automatically counted with a BZ-X700 microscope (KEYENCE) and its installed software, using at least 5 randomly selected views for each sample. The results are expressed as the mean ± standard deviation (SD) for each mouse.

### Statistical analysis

Continuous variables are reported as the mean ± SD and were compared using Student’s t-test or one-way analysis of variance with Tukey’s honestly significant difference test. P-values < 0.05 were considered statistically significant. All statistical analyses were performed using JMP Pro software, version 13.0 (SAS).

## Supplementary information

Supplemental information

## Data Availability

The authors confirm that the data supporting the findings of this study are available within the article and its supplementary materials.
