# Supplementary material for: Natural killer cells impede the engraftment of cardiomyocytes derived from induced pluripotent stem cells in syngeneic mouse model
Source: Sci Rep. 2019 Jul 25;9:10840. doi: 10.1038/s41598-019-47134-3 (PMC6658523; doi:10.1038/s41598-019-47134-3)
Supplement: Supplementary file 1 — Supplemental information [file 41598_2019_47134_MOESM1_ESM.pdf]

Figure S1

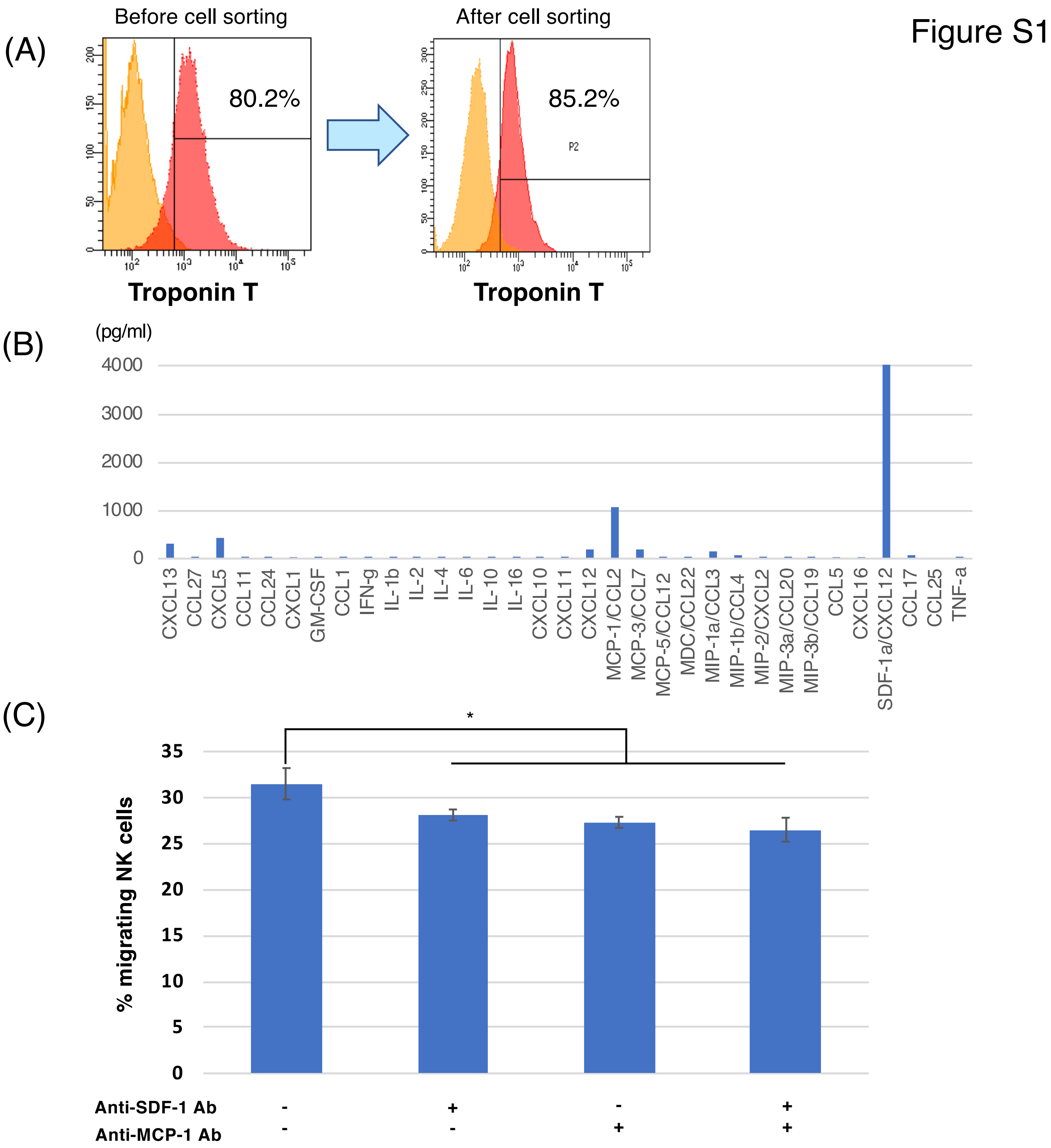

Figure S1. The purtiy of iPSC-CM after sorting and comprehensive analysis of chemokine secretion from iPSC-CM sheets. Related to Figure 1.

(A) Flow cytometry data for iPSC-CMs before and after eliminating SSEA-1 positive iPSC, stained with anti-troponin T antibodies or an isotype-matched control.

(B) In vitro screening for secreted chemokines in iPSC-CM sheet culture supernatants

(C) The percentage of fluorescence intensities of NKCs labeled with CAM compared with that of the control well, which NKCs added to the bottom wells. and expressed as the mean ± SD (n = 3, respectively). *$p < 0.05$

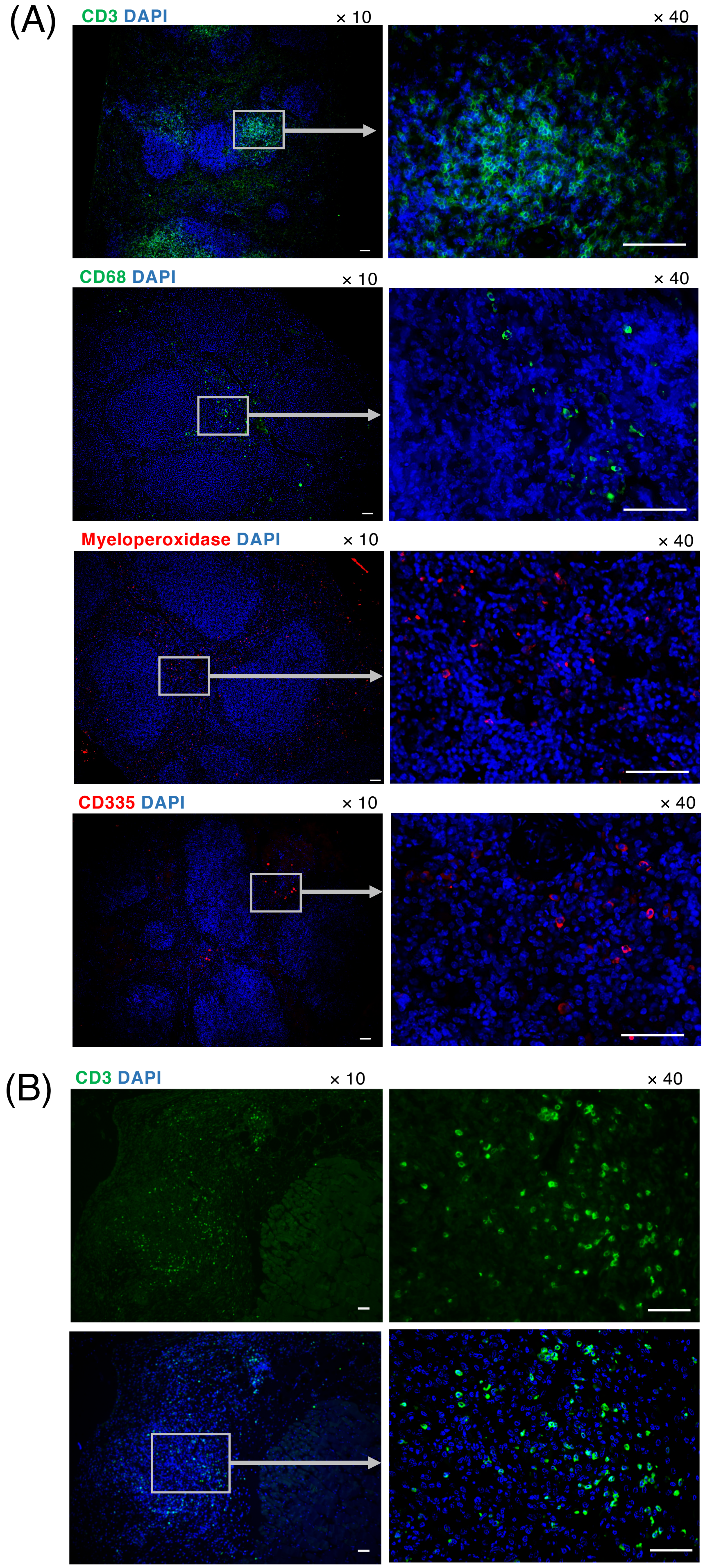
(A)
CD3 DAPI
× 10
× 40
CD68 DAPI
× 10
× 40
Myeloperoxidase DAPI
× 10
× 40
CD335 DAPI
× 10
× 40
(B)
CD3 DAPI
× 10
× 40

Figure S2

Figure S2. Positive controls for CD3, CD68, myeloperoxidase, and CD335 staining. Related to Figure 1

(A) C57BL/6 spleen stained with DAPI and antibodies against CD3 (Alexa Fluor 488), CD68 (Alexa Fluor 488), myeloperoxidase (Alexa Fluor 647), and CD335 (Alexa Flour 647). Scale bars: 50 μm

(B) Subcutaneous tissue 4 days after iPSC-CM allogeneic transplantation stained with anti-CD3 (Alexa Fluor 488), and DAPI. Scale bars: 50 μm.

Figure S3

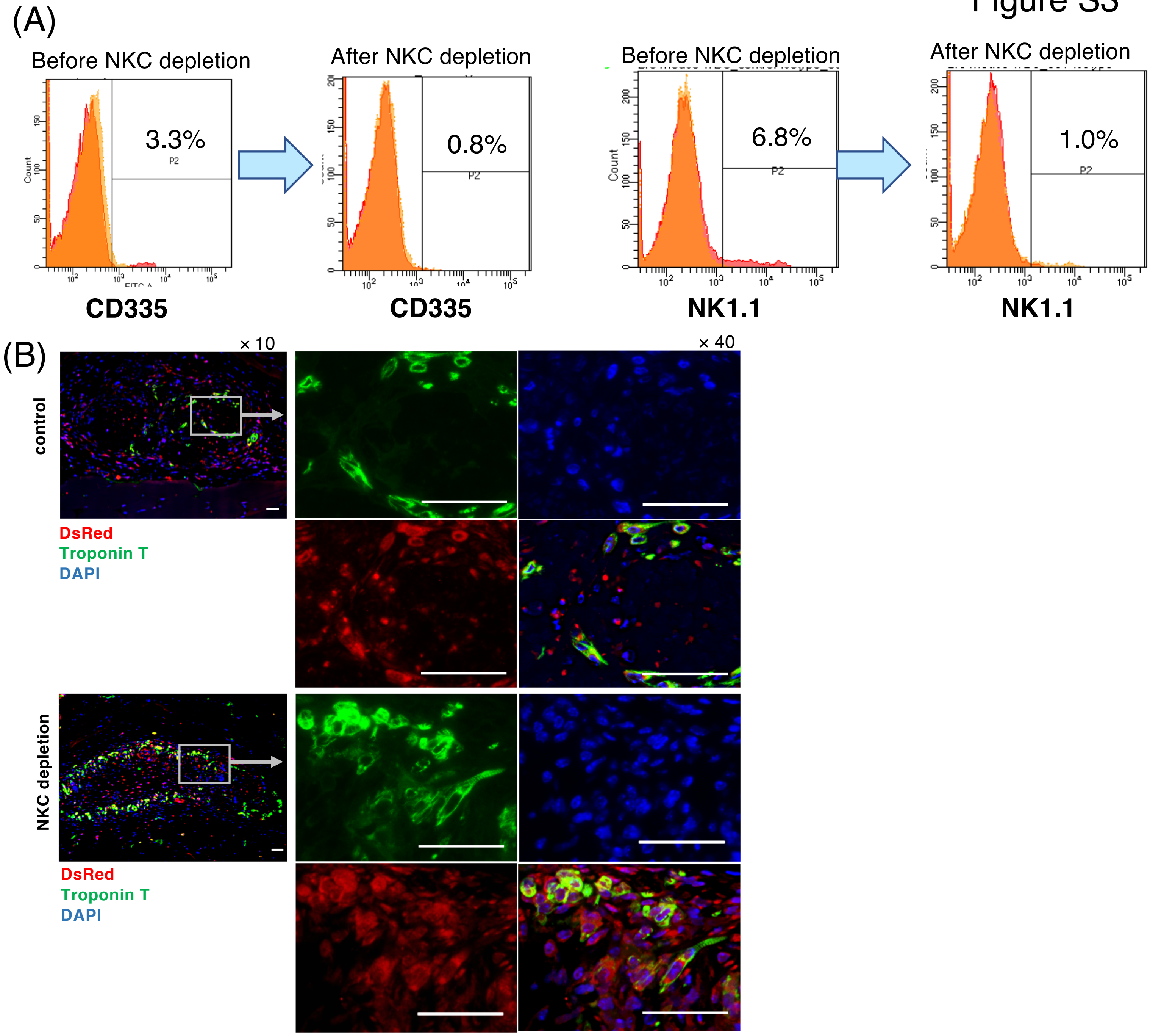

Figure S3. Establishment of an NKC-deleted model by administering an anti-NK1.1 antibody and histological analysis of survival cell 7days after transplantation. Related to Figure 2.

(A) Flow cytometric detection of NKC makers on splenocytes in a C57BL/6 mouse before and after administration of anti-NK1.1 antibodies, following stained with antibodies against CD335 and NK1.1, or isotype-matched control antibodies

(B) Staining of subcutaneous tissue with DAPI and an anti-RFP antibody (Alexa Flour 555) and anti-Troponin T (Alexa Flour 488) at 7 days post-iPSC-CM transplantation in an NKC-depleted model. Scale bars: 50 μm

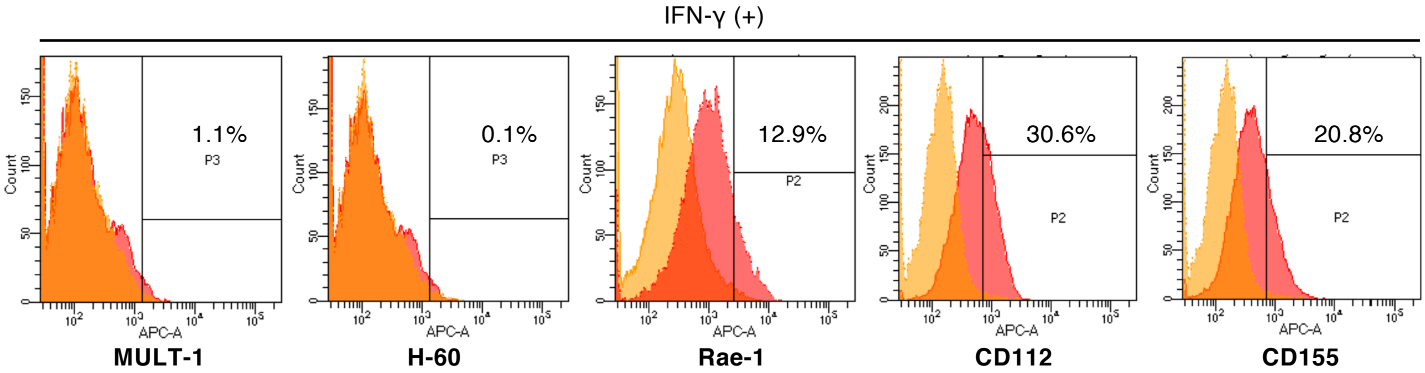

Figure S4. Expression analysis of activating NKC ligands on iPSC-CMs with IFN-γ treatment. Related to Figure 3.

Flow cytometric detection of NKC-activating ligands on iPSC-CMs at day 16 with IFN-γ treatment. The iPSC-CMs were stained with antibodies against MULT1, H-60, RAE-1, CD112, and CD155, or isotype-matched control antibodies.

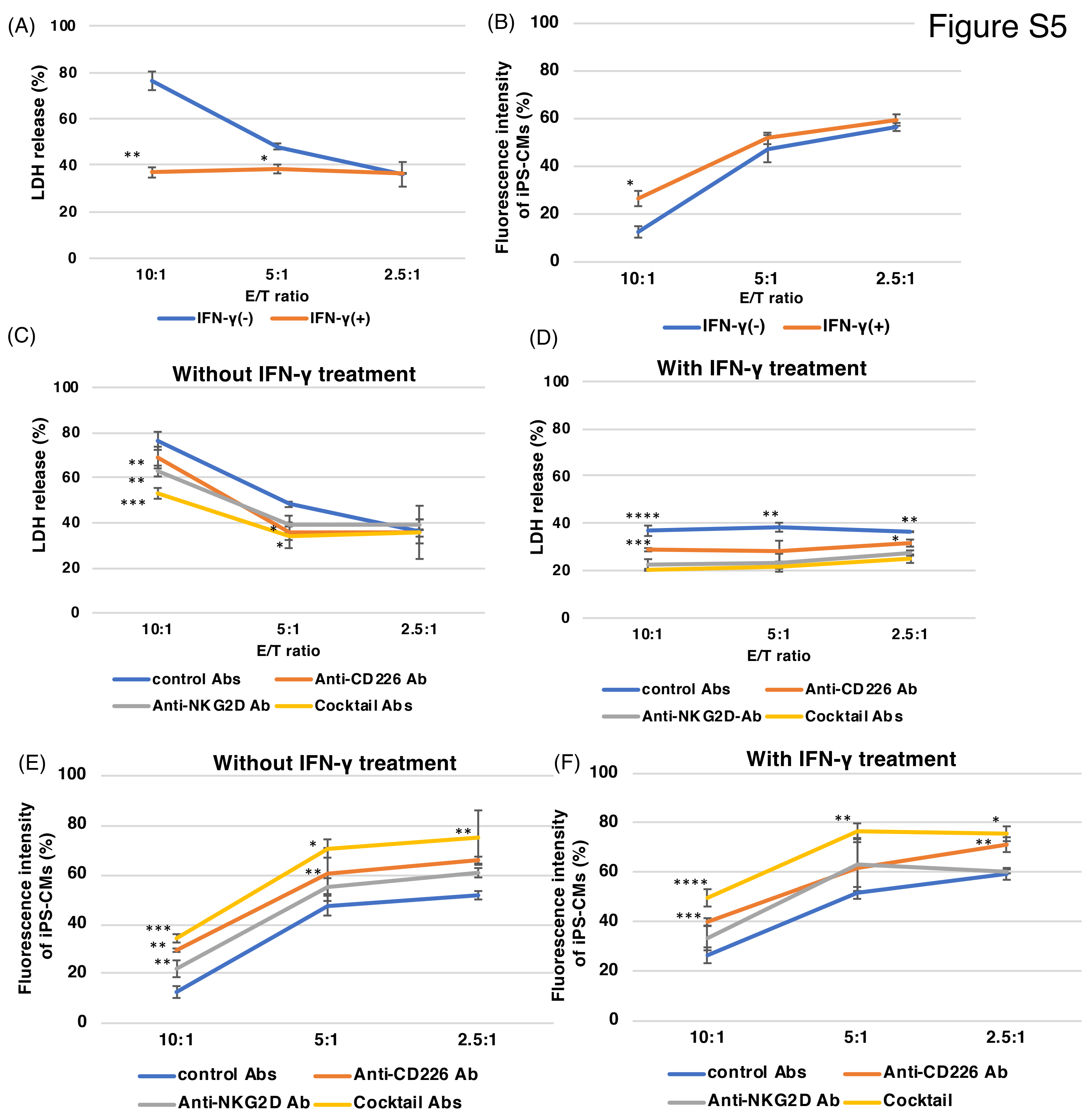
Figure S5
(A)
LDH release (%)
E/T ratio
IFN-γ(-)
IFN-γ(+)
(B)
Fluorescence intensity of iPS-CMs (%)
E/T ratio
IFN-γ(-)
IFN-γ(+)
(C)
Without IFN-γ treatment
LDH release (%)
E/T ratio
control Abs
Anti-CD226 Ab
Anti-NKG2D Ab
Cocktail Abs
(D)
With IFN-γ treatment
LDH release (%)
E/T ratio
control Abs
Anti-CD226 Ab
Anti-NKG2D-Ab
Cocktail Abs
(E)
Without IFN-γ treatment
Fluorescence intensity of iPS-CMs (%)
control Abs
Anti-CD226 Ab
Anti-NKG2D Ab
Cocktail Abs
(F)
With IFN-γ treatment
Fluorescence intensity of iPS-CMs (%)
control Abs
Anti-CD226 Ab
Anti-NKG2D Ab
Cocktail

FigureS5. LDH-release assay and Cell viability of iPSC-CMs after co-culture with NKCs. Related to Figure 4.

(A)The percentage of LDH release in supernatants of co-cultured iPSC-CMs and NKCs compared with that of 0.5% Triton X-treated controls and expressed as the mean ± SD (n = 3, respectively). **p < 0.0001, *p < 0.05 (vs iPSC-CM without IFN-γ treatment)

(B)The percentage of fluorescence intensities of iPSC-CMs labeled with CAM compared with that of 0.5% Triton X-treated control and expressed as the mean ± SD (n = 3, respectively). *p < 0.0001 (vs iPSC-CM without IFN-γ treatment)

(C) The percentage of fluorescence intensities of iPSC-CMs labeled with CAM compared with that of 0.5% Triton X-treated control and expressed as the mean ± SD (n = 3, respectively). ***; p<0.01 (vs Anti-CD226 Ab, Anti-NKG2D Ab, and Control Abs), **; p<0.0001 (vs Control Abs), *; p<0.05 (vs Control Abs)

(D) The percentage of fluorescence intensities of IFN-γ-treated iPSC-CMs labeled with CAM compared with that of 0.5% Triton X-treated control and expressed as the mean ± SD (n = 3, respectively). ****; p<0.0001 (vs Anti-CD226 Ab, Anti-NKG2D Ab, and Cocktail Abs), ***; p<0.01 (vs Cockatil Abs), **; p<0.05 (vs Anti-CD226 Ab, Anti-NKG2D Ab, and Cocktail Abs), *; p<0.05 (vs Cocktail Abs)

(E) The percentage of fluorescence intensities of iPSC-CMs labeled with CAM compared with that of 0.5% Triton X-treated control and expressed as the mean ± SD (n = 3, respectively). ***; p<0.001 (vs Anti-CD226 Ab, Anti-NKG2D Ab, and Control Abs), **; p<0.05 (vs Control Abs), *; p<0.05 (vs Anti-NKG2D Ab, and Control Abs)

(F) The percentage of fluorescence intensities of IFN-γ-treated iPSC-CMs labeled with CAM compared with that of 0.5% Triton X-treated control and expressed as the mean ± SD (n = 3, respectively). ****; p<0.01 (vs Anti-CD226 Ab, Anti-NKG2D Ab, and Control Abs), ***; p<0.001 (vs Control Abs), **; p<0.05 (vs Control Abs), *; p<0.01 (vs Anti-NKG2D Ab, and Control Abs)
